# Fluctuations in anthropogenic nighttime lights from satellite imagery for five cities in Niger and Nigeria

**DOI:** 10.1038/sdata.2018.256

**Published:** 2018-11-13

**Authors:** Nita Bharti, Andrew J. Tatem

**Affiliations:** 1 Department of Biology, Center for Infectious Disease Dynamics, Penn State University Pennsylvania, PA 16802, USA; 2 WorldPop, Department of Geography and Environment, University of Southampton; Flowminder Foundation, Southampton, SO17 1BJ, UK

**Keywords:** Population dynamics, Geography

## Abstract

Dynamic measures of human populations are critical for global health management but are often overlooked, largely because they are difficult to quantify. Measuring human population dynamics can be prohibitively expensive in under-resourced communities. Satellite imagery can provide measurements of human populations, past and present, to complement public health analyses and interventions. We used anthropogenic illumination from publicly accessible, serial satellite nighttime images as a quantifiable proxy for seasonal population variation in five urban areas in Niger and Nigeria. We identified population fluxes as the mechanistic driver of regional seasonal measles outbreaks. Our data showed 1) urban illumination fluctuated seasonally, 2) corresponding population fluctuations were sufficient to drive seasonal measles outbreaks, and 3) overlooking these fluctuations during vaccination activities resulted in below-target coverage levels, incapable of halting transmission of the virus. We designed immunization solutions capable of achieving above-target coverage of both resident and mobile populations. Here, we provide detailed data on brightness from 2000–2005 for 5 cities in Niger and Nigeria and detailed methodology for application to other populations.

## Background & Summary

Human populations are difficult to measure; their dynamics are even more difficult to measure. Populations are fluid by nature but are classically represented by static census-derived values. While censuses are extremely effective for measuring long-term changes and trends in populations, they are expensive and may be conducted at irregular intervals where resources are strained; population sizes for non-census years are extrapolated, often with linear annual growth rates. These estimates contain high levels of uncertainty but are used to assess public health needs and risks. Censuses are not designed to capture intra-annual changes in population size and are unable to do so.

**Figure 1:**
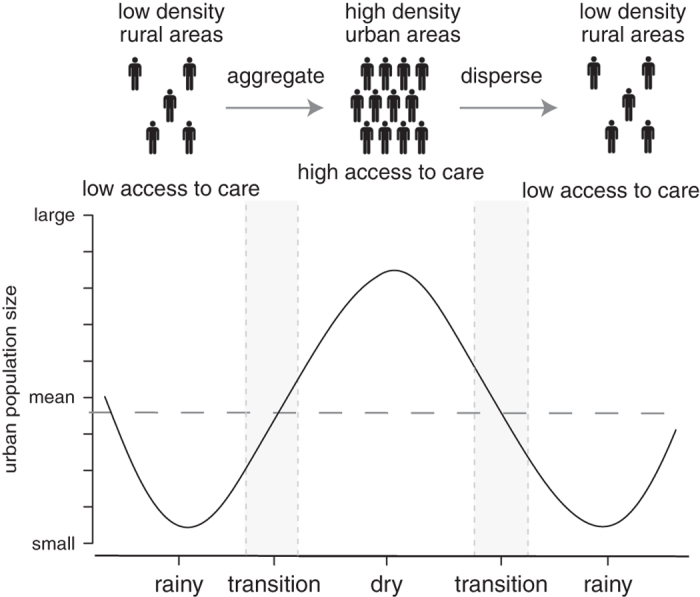
Schematic of population dynamics for seasonally mobile populations. In agriculturally driven economies, such as Niger, population density, measles transmission, and access to health care simultaneously increase during the dry season as urban populations grow. These populations decline during the rainy season, leading to decreased measles transmission as well as reduced access to health care and measles prevention.

Decadal or annual population measurements or projections are often insufficient and inappropriate for public health and epidemiologic interventions^1^. Target population sizes can change drastically within relevant temporal and spatial scales and population dynamics can drive important health and disease dynamics^2^. Specifically, changing population sizes alter contact rates and disease transmission^3,4^. Population dynamics also modify necessary health care capacity and disease prevention needs (Fig. 1)^5^. Human movement and migration constantly impact public health priorities^6^, disease ecology interactions^7^, and access to health care^1,8^. Environmental, economic, or agricultural cycles^9^, political instabilities, conflicts^10^, climate change, natural disasters^11^, or ongoing health events can drive human movements^12^. Vaccine-preventable diseases have persisted in areas where population movements are poorly understood, highlighting their importance. In other words, a failure to account for population movements is at least partially responsible for the low efficacy of specific public health measures in many areas.

Recurring seasonal migration is common in agriculturally driven economies, including areas throughout West Africa^13^, with impacts on transmissible diseases^14^. Rural-urban migration cycles can cause seasonal swells in urban population sizes when agricultural labor opportunities in rural areas are limited. Dynamic population sizes influence communicable diseases, first by changing contact rates, which drive disease transmission^15^, and second by introducing or dispersing pathogens via immigration and emigration^16^. As populations increase seasonally, they include migrants with diverse immune backgrounds and varying access to immunizations and health care because, relative to rural areas, urban areas often have better access to health care services^17^. If urban health care facilities could reliably predict and quantify seasonal fluctuations in population sizes, they could respond with corresponding adjustments in health care capacity. This would provide an economical and efficient improvement in regional health care and immunization coverage. This strategy would be particularly impactful for migrating populations who otherwise reside in areas that are difficult to reach for public health campaigns, including immunizations, and where health care facilities are lacking^17^. Urban health care facilities that prepare for population fluctuations may also avoid becoming overburdened in the face of increased seasonal demand or seasonal outbreaks. These efforts could reduce the overall prevalence and risk of transmissible diseases across regional metapopulations.

**Figure 2:**
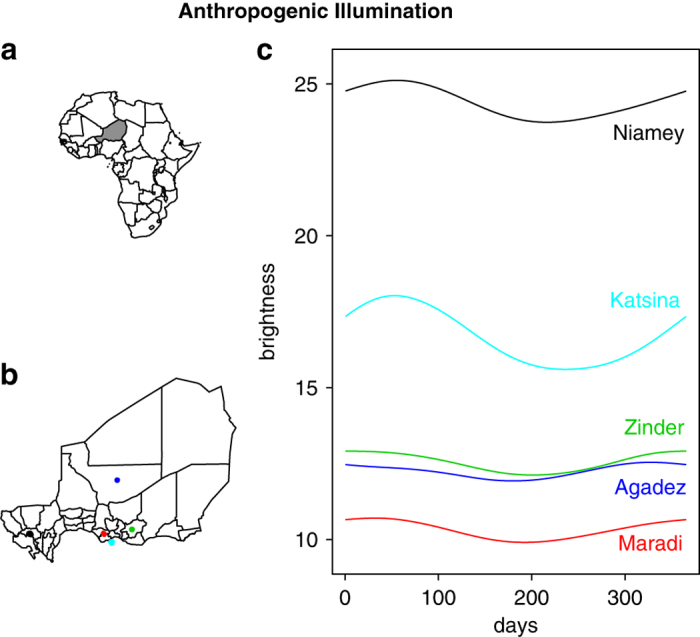
Anthropogenic illumination by city. (**a**) Map of Africa with Niger shaded. (**b**) Map of Niger showing locations of the five cities for which DMSP nighttime lights satellite imagery was analyzed for this study. Colors as in c. (**c**) Smoothed annual signature of mean brightness for each city.

We focused on five West African cities, four in Niger and one in northern Nigeria, near their shared border (Fig. 2a,b). Niger is at the edge of the Sahara Desert where seasonal rural-urban agricultural migration is common. Labor prospects are dispersed throughout agricultural areas surrounding the cities during the rainy season, which occurs from around June to September, and shift to the cities during the dry season, from around November to March. A decade of monthly measles case records from Niger’s public health districts show that measles outbreaks in Niger occur only during the dry season^18^. Although population fluctuations had long been a suspected contributing factor, they had never been quantified and were thought to be impossible to measure. We worked with the Ministry of Health of Niger and *Medecins sans Frontiers* and their collection of urban, weekly, spatially explicit measles case reports across an 11-year period to develop a method to measure populations in urban areas at epidemiologically relevant spatial and temporal resolution. We quantified nighttime brightness to show that urban illumination, a proxy for population size, fluctuated seasonally, and these fluctuations were sufficient to drive measles outbreaks^4^. We also showed that reactive vaccination campaigns occurred when urban brightness curves were near peak annual values but dose distribution targets were not adjusted for these fluxes^5^. Instead, the number of doses distributed in urban populations was calculated for permanent residents without accounting for increases due to seasonal migrants.

Here, we detail the methods and data underlying these findings: increases in urban brightness for five cities in Niger and Nigeria from 2000–2004 consistently occurred during the dry season and coincided with seasonal measles outbreaks from 1995–2005^4,5^ (Fig. 2c). We include a comparative analysis of brightness fluctuations between the communes within Niamey, Niger’s capital. These data provided a quantifiable, mechanistic explanation for Niger’s seasonal measles outbreaks.

## Methods

**Figure 3:**
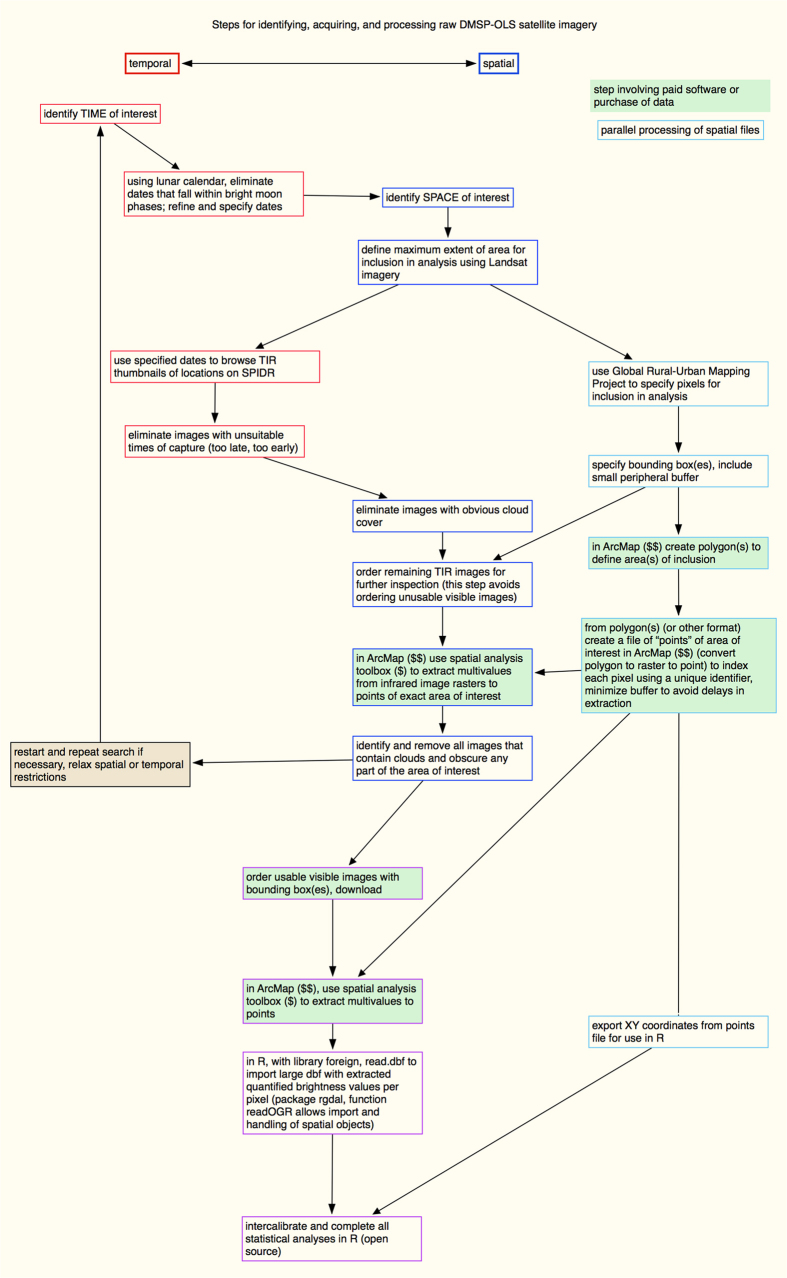
Workflow for the process of measuring human population dynamics with anthropogenic illumination, as captured by satellite imagery DMSP-OLS. Temporally focused steps in the process are outlined in red, spatially focused elements are outlined in blue. Steps that integrate spatial and temporal data or analysis are outlined in purple. Boxes shaded in green indicate a step for which we use a paid resource, either software or data. We assume ArcGIS is not freely available and tasks for which we use it are shaded in green.

To measure seasonal population fluctuations, we used nighttime captured satellite images to quantify anthropogenic illumination, which is a direct representation of human presence^19^ (Fig. 3). Using serial satellite imagery, we quantified the daily changes in brightness per pixel and area lit. We measured fluctuations in brightness, a proxy for relative population size, from 2000–2005 across the four largest cities in Niger: the capital city, Niamey, as well as Maradi, Zinder, and Agadez. The northern uranium-mining city of Agadez in Niger was included for contrast because the economy is not solely agriculturally driven. We also included the northern Nigerian city Katsina, which is very close to the Niger border, has significant interactions with southern Niger^20^, and displays the same seasonal measles dynamics as the agriculturally influenced cities in Niger.

### Specifications of Satellite Imagery Used

Images were obtained from the Department of Defense (DoD) program called the Defense Meteorological Satellite Program’s (DMSP) Operational Linescan System (OLS), which is run by the Air Force Space and Missile Systems Center (SMC). The data are sent to National Oceanographic and Atmospheric Administration’s (NOAA) National Centers for Environment Information (NCEI; formerly known as National Geophysical Data Center, NGDC). We accessed the images through the Space Physics Interactive Data Resource (SPIDR, formerly http://spidr.ngdc.noaa.gov/spidr/ now http://spidr.ionosonde.net/spidr/)^21^ and used 155 images captured by the F15 sensor, which provided usable imagery from 2000–2007.

DMSP OLS satellites orbit at 833 km and simultaneously capture two broad spectral bands; a visible band (0.40–1.10 μm) and an infrared band (10.0–13.4 μm) (more details: https://ngdc.noaa.gov/eog/sensors/ols.html). Visible images display nighttime brightness and the infrared band detects cloud cover (details below). Newer satellites capture similar data at higher spatial resolution but do not provide reliable data prior to 2012 (for example, the Visible Infrared Imaging Radiometer Suite (VIIRS)^22^). Historical baseline measurements of population dynamics, like those provided by DMSP OLS images, are invaluable for calibrating newer data streams and understanding long term changes in settlement patterns.

### Temporal Specification

We focused our analysis on four cities in Niger and one city in Nigeria, using images captured during 2000 and 2002–2004. We chose these dates because we were investigating weekly measles cases recorded during this time frame for each of the cities in Niger. We did not use images from 2001 because it was an exceptionally cloudy year. Although our analysis included one city from Nigeria, we do not have measles reports for any Nigerian locations.

To minimize brightness fluctuations from changes in human behavior, we used images that were captured at approximately the same time every day, focusing on the hours between 7 and 10 pm in local time. DMSP OLS images have a naming convention that indicates the date and GMT time of capture so local time can be calculated accurately. The sensor is indicated in the first three characters of the image name (see more below). Images are named as follows:

FXXYYYYMMDDHHMM

FXX = sensor

YYYY = year in four digit format

MM = month in two digit format

DD = date in two digit format

HH = GMT hour in two digit format

MM = GMT minute in two digit format

### Spatial Specification

The maximum spatial extent of each urban settlement analyzed in this study was defined using Landsat imagery from 2000 and 2005 (details in^4^ supporting online material). We included all the pixels that were identified in the Global Rural-Urban Mapping Project (GRUMP)^23,24^, which relied on DMSP OLS composite data across multiple years. In using this approach, some of the mapped urban extents extended beyond the formal administrative boundaries of a city. This was caused either by an urban population that expanded beyond the formal boundaries of a city or the “overglow” effect in which lighting produced in one location bloomed to an adjacent area^25^. In case of the former, it was important to include these areas; in the event of the latter, the relative brightness values would indicate valid increases or decreases. We held the spatial extent of each urban area constant throughout the study. We outlined each urban area included in this study with a polygon, we then we converted that polygon to points in ArcGIS^26^. This created an indexed pixel file for each urban spatial extent in which each pixel was given a unique, numeric identifier that corresponded to the pixel’s exact location by latitudinal and longitudinal coordinates. We refer to this as a ‘pixel file’ (columns A-D in ntl.cal155.brt.csv, Data Citation 1).

We used spatial data provided by *MSF* to define the spatial extent of each of the three communes of Niger’s capital city, Niamey, as they were designated in 2004 (ntl.niameypixcomm.csv, Data Citation 1).

### Image selection refinement and ordering

Images are captured nightly but not all images are usable; solar and lunar illumination can contaminate brightness values in these images. We identified and removed evening images captured before sunset and during bright moon phases using a lunar calendar (stardate.org), which provided daily lunar phase data^19^.

Cloud cover also obstructs visible satellite imagery. The thermal band captures Thermal Infrared (TIR) images, which allow us to identify areas of cloud cover because they are colder than cloud-free areas; NOAA executed their standard in-house cloud-screening algorithm on the TIR files associated with our candidate images^27^. The algorithm compared the OLS thermal band to modeled surface temperature values. If the OLS thermal band recorded data significantly colder than the modeled surface temperature values, the algorithm suggests this may be cloud cover. NOAA does not guarantee that these results identify cloud cover with 100% accuracy but they offer their cloud screening algorithm as a loose guide for selecting cloud-free images.

We also extracted the digital TIR value of each urban pixel in each image using “Extract multivalues to points” (in ArcGIS ArcMap: Spatial Analysis Toolbox - > Extraction). The output values from this process further alerted us to the presence of clouds over the pre-defined pixels of interest. Values greater than 50 exceeded our threshold for cloud tolerance and those images would be discarded from the analysis.

After this preliminary image selection was complete, we obtained TIR files of specified spatial extents for the images we had approved up to this point. TIR geotiffs were obtained at no charge and allowed us to obtain highly detailed cloud locations before purchasing visible images. We visually inspected each of the remaining candidate TIR images and independently assessed each image for cloud cover over the cities of interest. These rankings were used to further eliminate cloudy images as detailed below.

Each of the two authors ranked each TIR image for cloud cover over the pixels of each city on a scale from 1-5 of cloudy to clear. Each author was blind to the other’s rankings; final rankings were then compared. Images that were ranked 4 or 5 by both authors were considered cloud free and were approved for inclusion in the analysis. Any images receiving two rankings of 3 or below were discarded from further consideration. Images with one 4 or 5 ranking and one 3 ranking were reconsidered and the authors collectively decided whether the image qualified for inclusion in the analysis. No images receiving a 4 or 5 from one author received a ranking lower than 3 from the other author. Overall, cloud rankings were + /− 1 between the authors across all TIR images, showing high agreement.

We used the resulting list of cloud-free TIR images to order the simultaneously captured visible images over our spatial extent; for TIR images with cloud presence over pixels of interest, the paired visible image was not included in our analysis.

### Analysis of visible images

We visually inspected each visible image and discarded images that were either totally dark (no value for any pixels, indicating errors in capture or processing and easily distinguished from a blackout in the city), displayed a sun glare, or were blurry (indicating an error in sensor speed correction). We extracted the pixel-level brightness values from all the remaining images in ArcGIS. Using a pixel file for each urban area, we used the Spatial Analysis Toolbox, the Extraction tool, and the selection Extract Multi-values to points. This extracted pixel-level brightness values from batches of visible images and wrote the numerical brightness value of each point to the correct location in the pixel file.

We calibrated the brightness values of each pixel using intercalibration equations and parameters previously published by Elvidge, *et al.*^28,29^ (see also Usage Notes below). We arranged four years of satellite imagery into a single annual signal, ordering the images by the day of capture (ntl.cal155.brt.csv, Data Citation 1). We created a matrix where three annual patterns of brightness by pixel were pasted together and we fit a smoothing spline to the values for each pixel. We used the central 365 values for each pixel for visualization purposes, allowing us to inform the terminal points with the preceding and following dates of the annual cycle (ntl.calsmth365.brt.csv, Data Citation 1; ntl.animation.mp4, Data Citation 1).

### Software Used

To define spatial extents, create pixel files, and extract cloud temperatures and brightness values, we used ArcGIS versions 9 and 10 and the spatial analyst toolbox, which we purchased^26^. ArgGIS and various toolboxes are available at many Universities and institutions that have purchased group licenses. There are also free spatial analysis software alternatives.

All statistical analyses were completed in R version 2.5, which is an open access and freely available software environment, available for download here https://www.r-project.org^30^. In R version 2.5, we used the package maptools and the function readShapeSpatial to import and analyze spatial objects in R. R 3.0 and later versions no longer support the readShapeSpatial function; those versions can use the package rgdal to manage spatial objects in R (ntl.code.R, Data Citation 1 and Fig. 3). We used the package foreign and the function read.dbf, to read dbf files into a data frame in R; this can also be done by converting dbf files to csv files and using read.csv (ntl.code.R, Data Citation 1). Packages and their documentation can be downloaded here: https://cran.r-project.org/web/packages/available_packages_by_name.html.

## Data Records

All data described in the article are available publicly and freely through ScholarSphere (Table 1). They will be preserved in their published form.

## Technical Validation

The quantifiable value of nighttime lights brightness as a direct indicator of human presence and population size has previously been validated^31^. The relationship between population dynamics and nighttime lights dynamics has also been validated^29^. Although these dynamics were primarily studied across longer time scales using annual composites instead of individual images, the same underlying principles apply across shorter time scales. We also have preliminary results showing that a similar analysis we conducted using more recent, higher resolution nighttime lights imagery from VIIRS displays seasonal fluctuations in anthropogenic illumination cycles for these five cities that match the ones detected using these DMSP-OLS data.

We demonstrated that four cities with agriculturally driven economies experienced brightness fluctuations that reflect seasonal labor migration (Fig. 2c). We also showed that Agadez, a city with an agriculturally influenced economy that is dominated by uranium mining, had weaker fluctuations that were slightly delayed when compared to the other cities^4^. This indicates that these images are capturing real fluctuations in cities and the detected fluctuations in brightness are not likely to be artifacts. Using these fluctuations to calculate the number of vaccine doses that must be administered to a population to halt measles transmission at the specific time of the intervention helps reduce urban, seasonal outbreaks and increase regional vaccine coverage^5^.

At higher spatial resolution, we also showed that commune 3 in the city of Niamey, Niger, showed dampened seasonal fluctuations in brightness, which lagged the other two, larger communes of the city^4^ (ntl.niameypixcomm.csv, Data Citation 1). The phenomenological reason for the observed delayed and dampened fluctuations in the third commune of the city is likely due to economic and demographic differences in that part of the city. Observing and quantifying the distinct pattern is important because it clearly demonstrates that the imagery can capture differences not only between cities, but also within a city. Light blooms or overglow effects did not conceal the light patterns of the communes within Niamey.

The passive surveillance of satellites provides an opportunity to gather data from the past. Passive surveillance data streams can provide essential ‘baseline’ measurements to help interpret data collected after an event or disturbance. The spatial and temporal reach of satellites surpasses most data collection systems. The DMPS OLS nighttime lights imagery provides daily global coverage of visible brightness, dating back to 1992. These data are useful for interpreting new satellite imagery streams. While today’s satellite missions can collect anthropogenic brightness values and hyperspectral data at higher spatial resolution and with greater sensitivity, they cannot collect data prior to their launch dates. The methods and data presented here allow scientists to create a baseline of satellite data of visible light and thermal cloud patterns.

We integrate NOAA’s DMSP nighttime lights imagery with other data sources, as detailed in the methods section, to create this novel data product. NOAA’s easily downloadable, annual composite products are an excellent resource but they allow only for comparisons *between* years. We serially compiled NOAA’s less-accessible, daily, raw, visible light imagery to quantify seasonal changes in anthropogenic illumination, which can detect population fluctuations *within* each year. This permits analysis at a flexible time scale that is highly relevant to disease transmission and prevention, and can be applied to a number of other global health and humanitarian efforts. Prior to our development of these methods, seasonal anthropogenic illumination had not been quantified.

### Broader usage and applications

Anthropogenic illumination signals can provide unique insight into the dynamics of human settlements, human movement and behavior, and human interactions with the environment. A proxy for long-term, global human settlement patterns at high spatial and temporal resolution is otherwise nearly impossible to obtain. Applications of these data include our previous uses of understanding infectious disease outbreaks^4^, planning vaccination strategies^5^, as well as assessing international displacement caused by political instability^32^. Broader applications of these data and methods for the scientific community extend to natural disaster and emergency response efforts, characterizing routine movement patterns and their disruptions due to climate change, measuring large-scale displacement and locating refugee populations, and many more. These data provide valuable information on the past, present, and future seasonal patterns of human settlements.

Importantly, data on brightness values represent the vast majority of individuals within a population because they include electrification and fires. Newer technologies, such as mobile phone call detail records^33^, geotagged social media posts^34^, Google location history^35^, and other mobile phone based locational services, may only have situational utility or limited representation of populations. These data tend to underrepresent rural populations^36^, specific demographics within a population^37^, or low income and under-resourced individuals^38^. Additionally, the use of newer technologies to track human movements, such as the previously mentioned mobile phone derived data streams, are restricted to dates that align with the widespread adoption of those technologies, which is a very recent or ongoing process in many areas of global health interest. The data provided here can be paired with newer technologies to understand long-term spatial patterns of human settlements. Finally, many data derived from new technologies are privately owned and cannot be shared within the scientific community. Nearly all studies that use proprietary data, such as mobile phone call detail records or mobility traces cannot be replicated because the data cannot be shared. These publicly available satellite data and methods can assist scientists working with privatized data to augment their confidential datasets. Such efforts can help scientists across disciplines assess data accuracy and improve data representation, addressing a major challenge in global health.

## Usage notes

### Calibration

Sensors can drift and degrade over time in orbit and no two are identical. To ensure compatibility of images captured by more than one sensor or across multiple years, we used intercalibration equations and parameters developed by Elvidge *et al.*^28,29^, we completed this step in R.

### Sensor saturation and sensitivity

Quantifying nighttime anthropogenic illumination to assess changes in population size is best suited for urban areas in low-income nations. Non-urban areas in low-income nations may be too dimly lit for detection in images^27^. Conversely, most urban and suburban areas in high-income nations are too bright to detect increasing levels of brightness^27^. In this case, the saturated sensor would report maximum brightness even if the city increased in illumination.

## Additional information

**How to cite this article**: Bharti, N. *et al*. Fluctuations in anthropogenic nighttime lights from satellite imagery for five cities in Niger and Nigeria. *Sci. Data*. 5:180256 doi: 10.1038/sdata.2018.256 (2018).

**Publisher’s note**: Springer Nature remains neutral with regard to jurisdictional claims in published maps and institutional affiliations.

## Supplementary Material



## Figures and Tables

**Table 1:** Data records for all associated data files available via ScholarSphere.

**File Name**	**Description**	**Format**	**Data Citation**	DOI
ntl.cal155.brt.csv	calibrated, unsmoothed brightness values for 155 days, 925 pixels	csv	1	https://doi.org/10.18113/S1XH02
ntl.calsmth365.brt.csv	calibrated smoothed daily brightness values for 365 days, 925 pixels	csv	1	https://doi.org/10.18113/S1XH02
ntl.niameypixcomm.csv	pixel location, ID, commune designation within Niamey	csv	1	https://doi.org/10.18113/S1XH02
ntl.animation.mp4	animation of nighttime lights for each city^39^	mp4	1	https://doi.org/10.18113/S1XH02
ntl.code.r	R code to plot Fig. 2, additional plots	text	1	https://doi.org/10.18113/S1XH02
